# SARS-CoV-2-specific immunoglobulin Y antibodies are protective in infected mice

**DOI:** 10.1371/journal.ppat.1010782

**Published:** 2022-09-19

**Authors:** Sherif A. El-Kafrawy, Abby Odle, Aymn T. Abbas, Ahmed M. Hassan, Umama A. Abdel-dayem, Arooj K. Qureshi, Lok-Yin Roy Wong, Jian Zheng, David K. Meyerholz, Stanley Perlman, Alimuddin Zumla, Esam I. Azhar

**Affiliations:** 1 Special Infectious Agents Unit-BSL3, King Fahd Medical Research Center, King Abdulaziz University, Jeddah, Saudi Arabia; 2 Department of Medical Laboratory Sciences, Faculty of Applied Medical Sciences, King Abdulaziz University, Jeddah, Saudi Arabia; 3 Department of Microbiology and Immunology, University of Iowa, Iowa City, Iowa, United States of America; 4 Biotechnology Research Laboratories, Gastroenterology, Surgery Centre, Mansoura University, Mansoura, Egypt; 5 Animal Facility Unit, King Fahd Medical Research Center, King Abdulaziz University, Jeddah, Saudi Arabia; 6 Department of Pathology, University of Iowa, Iowa City, Iowa, United States of America; 7 Department of Infection, Division of Infection and Immunity, Centre for Clinical Microbiology, University College London, London, United Kingdom; 8 NIHR Biomedical Research Centre, University College London Hospitals, London, United Kingdom; Johns Hopkins Bloomberg School of Public Health, UNITED STATES

## Abstract

Safe, passive immunization methods are required against severe acute respiratory syndrome coronavirus-2 (SARS-CoV-2) and its variants. Immunization of chickens with antigen is known to induce specific IgY antibodies concentrated in the egg yolk and has a good safety profile, high yield of IgY per egg, can be topically applied, not requiring parenteral delivery. Our data provide the first evidence of the prophylactic efficacy of Immunoglobulin Y antibodies against SARS-CoV-2 in mice. Lohmann hens were injected with recombinant SARS-CoV-2 RBD protein; IgY-Abs were extracted from the eggs and characterized using SDS-PAGE. Antiviral activity was evaluated using plaque reduction neutralization tests. In additional experiments, IgY-RBD efficacy was examined in mice sensitized to SARS-CoV-2 infection by transduction with Ad5-hACE2 (mild disease) or by using mouse-adapted virus (severe disease). In both cases, prophylactic intranasal administration of IgY-Abs reduced SARS-CoV-2 replication, and reduced morbidity, inflammatory cell infiltration, hemorrhage, and edema in the lungs and increased survival compared to control groups that received non-specific IgY-Abs. These results indicate that further evaluation of IgY-RBD antibodies in humans is warranted.

## Author summary

We report the generation, characterization and antiviral activity of egg yolk derived IgY-Abs against the SARS-CoV-2 Receptor Binding Domain. Using two different models of murine SARS-CoV-2 infection, we show that intranasal prophylactic administration of IgY-Abs reduced viral replication, and reduced inflammatory cell infiltration, hemorrhage, and edema in the lungs.

## Introduction

Safe, cost-effective, universally available passive immunization methods are required to protect against infections caused by severe acute respiratory syndrome coronavirus-2 (SARS-CoV-2) and its variants. Immunoglobulin Y (IgY) is a primary antibody in the egg yolk of oviparous animals [1] and can be readily isolated using precipitation techniques [2]. Immunization of chickens with antigen leads to specific IgY antibodies accumulating in the egg yolk. IgYs have drawn considerable attention as potential alternatives to sera and other immunoglobulins for passive immunization [3–5]. IgYs are safer than IgGs because they do not bind to human Fc receptors or fix mammalian complement components; hence they do not trigger potentially dangerous immune responses [6]. Hakalehto et al 2021 reported that IgY antibodies are one of the safest possible therapeutic agents [7]. IgY consumed orally is considered to be GRAS (“Generally Recognized as Safe”) by the U.S. Food and Drug Administration [8]. Additionally, oral IgY antibodies have been applied to treat pulmonary *Pseudomonas aeruginosa* infected patients, and no negative side have been observed in up to 10 years of use [9].

Passive immunization with anti-SARS-CoV-2 IgY has several advantages including: a good safety profile, applicability to people in all geographical regions, high yield of IgY per egg, topical rather than injectable application, and rapid mass production at a low cost given the availability of large scale egg production for human consumption [10,11]. In addition, anti-SARS-CoV-2 IgY applied superficially to mucous membranes would not be expected to elicit antibody-dependent enhancement of infection [8]. Anti-SARS-CoV-2 IgY may be ideal for effective transient immunization while awaiting global COVID-19 vaccination or in immunocompromised cases where vaccines might not be effective. Administration of the anti-SARS-CoV-2 IgY to the nasal passage and throat mucosa through the intranasal route is thought to increase the efficacy as it is given to the viral entry and replication site [8]. This route of administration might provide immediate and short-lived protection in healthcare and other high-risk individuals in regions where new variants of concern might emerge due to vaccination or in areas where COVID-19 vaccination is unavailable [8]. Anti-spike-S1 IgY has been shown to neutralize SARS-CoV-2 *in vitro* and/or to prevent binding to the ACE2 (angiotensin converting enzyme 2) receptor on human cells [12–14]. The receptor-binding domain (RBD), a functional domain in S1 [15], binds to ACE2 enabling virus entry into cells [16–18]. Blocking RBD–ACE2 interactions can block cellular entry of SARS-CoV-2. Previously, we reported the use of IgY antibodies against the S1 and S proteins of MERS-CoV for efficient viral inhibition both *in vitro* and *in vivo* [4,19]. In this study, we generated and characterized IgY-antibodies targeting SARS-CoV-2 RBD. The *in vitro* and *in vivo* properties of these antibodies were evaluated using susceptible cell lines and two SARS-Cov-2 infected mouse models.

## Materials and methods

### Ethics and IRB statement

Chicken experimental protocols were reviewed and approved by the Institutional Animal Care and Use and CEGMR bioethics committee of King Fahd Medical Research Center., King Abdulaziz University (Permit No: 16-CEGMR-Bioeth-2021). Mouse experimental protocols were approved by the Institutional Animal Care and Use Committee of the University of Iowa, approval Number: 9051795

### Immunization of laying hens

Twenty-five weeks old Lohmann laying hens (12 hens) from a local farm (Fakieh Poultry Farms, Saudi Arabia) were used for egg production. Hens were housed in broiler chicken cages in a 12-h dark-light-cycle at a temperature of 24 ± 3°C. Commercial laying hen food and water are provided ad libitum. Hens were divided into two groups, the immunization group and the control group. Hens were immunized by injecting 200 μg of recombinant SARS- CoV-2 RBD protein purchased from Sino Biological, Inc. (Beijing, China) in the left or right side of the pectoral muscle on weeks 0, 2 and 4. Recombinant proteins were emulsified in a 1:1 ratio with Freund’s Complete Adjuvant (Sigma, St. Louis, MO, USA) for the first immunization and Freund’s Incomplete Adjuvant (Sigma, USA) for booster immunizations. The protein adjuvant mixture was pipetted up and down using a 5-ml syringe with a 19-gauge needle until stable. Hens of the control group (n = 6) were injected with a mixture of the corresponding adjuvant diluted in phosphate-buffered saline (PBS). Eggs were collected on daily basis starting 1 week before first immunization till 12 weeks post immunization and stored at 4°C until used for isolation of IgY.

### IgY extraction from chicken eggs

IgY were concentrated from eggs yolks using the polyethylene glycol 8000 (PEG 8000, Sigma, St. Louis, MO, USA) precipitation method [20] with minor modifications [21,22]. Eggs from each group were pooled weekly and the pooled yolk was used for IgY extraction. Egg yolks were briefly homogenized with three volumes of PBS (pH 7.4). Then PEG 8000 was added to a final concentration of 3.5%. The samples mixtures were then vortexed and rolled on rolling mixer for 20 min at room temperature. Subsequently, centrifugation of samples at 13,000 g for 20 min at 4°C removed the precipitated debris. The supernatant was passed through a folded filter and transferred to a new tube. After adjusting supernatants to 8.5% PEG 8000 and incubation for 15 min at room temperature, the samples were centrifuged again. Using the same method as above, the precipitated IgY pellets were suspended in 10 ml PBS, precipitated again with 12% of PEG 8000 and pelleted by last centrifugation. Finally, 2.0 ml PBS was added to the pellets and concentrated IgY were stored at -20°C after filtering through a 0.45 μm filters. The IgY concentration was determined using a NanoDrop 2000 spectrophotometer system (Thermo Scientific, USA).

### Sodium Dodecyl Sulfate–Polyacrylamide Gel Electrophoresis (SDS–PAGE)

To determine the molecular weight and purity of IgY, SDS–PAGE was performed using 12% PAGE in a Mini-PROTEAN 3 cell (Bio-Rad Laboratories, Hercules, CA, USA). SDS-PAGE was performed under reducing conditions where samples were boiled at 100°C with 2× sample buffer for 10 min, then purified IgY (25 μL) was loaded into each well with 1 well for molecular weight marker (prestained Blue Protein Marker, MOLEQULE-ON, Auckland, New Zealand). Electrophoresis was performed at room temperature in running Tris-glycine buffer at 200 volts for 40 min. Protein bands were visualized using Coomassie Brilliant Blue stain (Abcam, Cambridge, UK).

### Western Blot Assay (WB)

To confirm the specificity of the anti-SARS-CoV-2 RBD IgY antibody Western blot analysis was performed WB as previously described [23]. 500 ng of recombinant RBD protein was mixed with 20 μL of sample buffer, then subjected to SDS–PAGE at 200 V for 40 min at room temperature using a 14% polyacrylamide gel. One of the strips was stained with Coomassie Brilliant Blue stain (Abcam, Cambridge, UK) and used to determine the purity and size of the recombinant RBD protein by comparing to the molecular weight marker.

The PVDF membrane was activated by methanol then RBD protein was electrically transferred onto the membrane (Thermo Fisher, Waltham, MA, USA) at 30 V overnight. The membrane was divided into 0.5-cm wide strips and blocked for 1 h at room temperature with Tris-buffered saline containing 0.1% Tween 20 (TBS-T) and 5% non-fat milk. Strips were then washed three times for 10 min each then incubated in a 1:50 dilution of anti-SARS-CoV-2 RBD IgY antibodies. The strips were washed 3x 10 min with TBS-T then incubated for 1 h at room temperature with HRP-conjugated rabbit anti-chicken IgY Heavy and Light (Abcam, Cambridge, UK) at a 1:10,000 dilution in blocking buffer. The strips again were washed 3x 10 min, after which they were incubated for 15 min at room temperature with HRP colorimetric substrate (Immun-Blot Opti-4CN colorimetric Kit, Bio-Rad). The strips were rinsed with distilled water to stop the reaction. After band development, the strips were finally photographed.

### Reactivity of Anti-RBD IgY antibodies by ELISA

SARS-CoV-2 RBD antigen (Sino Biological, Inc., Beijing, China) were used to coat microtiter plates at a concentration of 500 ng/mL in PBS (0.01 M, pH 7.4) and incubated at 4°C overnight. Plates were washed and blocked using 250 μL of blocking buffer (5% skim milk in PBS-Tween) at room temperature for 1 h followed by washing three times with wash buffer. Purified IgY antibody samples from immunized and non-immunized hens were serially diluted starting from a 1:50 ratio in blocking buffer. Plates were then incubated at 37°C for 1 h and washed three times with PBS-Tween. Horseradish peroxidase (HRP)-conjugated rabbit anti-chicken IgY (Abcam, Cambridge, UK) at a 1:10,000 dilution was added in a 100 μL/well and incubated for 1 h at 37°C. Plates were washed and color was developed by adding 100 μL/well of TMB substrate solution (Promega, Madison, WI, USA) and incubating for 30 min. color development was stopped by adding 2M H_2_SO_4_ (100 μL/well).

Optical density (OD) was measured at a wavelength of 450 nm using ELISA plate reader (ELX800 Biokit) with PBS as a blank control and IgY extracted from non-immunized hens used as negative control. The IgY titer was defined as the maximum sample dilution that showed an OD value 2.1 times higher than that of the negative control.

### Plaque reduction neutralization test

Live virus experiments were performed in a biosafety level 3 laboratory of the Special Infectious Agents Unit, BSL-3 of King Fahd Medical Research Center at King Abdulaziz University in Jeddah or at the University of Iowa. Plaque reduction neutralization assays were performed to evaluate the neutralizing activity of anti-RBD IgY antibodies against SARS-CoV-2 as described [24]. Serial dilutions of IgY antibodies were incubated with an equal volume of 0.01 MOI SARS-CoV-2 or 60–70 plaques at 37°C for 30–60 min. Ancestral SARS-CoV-2 clinical isolates (SARS-CoV-2/human/SAU/85791C/2020, Genbank accession number MT630432.1; 2019-nCoV/USA-WA1/2020 SARS-CoV-2, Genbank accession number MN985325.1), and Delta (hCoV-19/USA/MD-HP05647/2021, BEI nr-55672) and Omicron (hCoV19/EHC_C19_2811C, obtained from Dr. Mehul Suthar, Emory University) variants were used for these assays. Subsequently, 200 μL of the incubated mixture were added to 95–100% confluent Vero E6 cells or VeroE6-TMPRSS2-hACE2 cells in 12-well plates and incubated at 37°C in 5% CO_2_ for 1 h, gently rocking every 10 minutes. Each assay included a cell control (PBS and cells) and a virus control (virus and cells). After incubation, the Vero cells were covered with agarose-containing overlay medium of 1.5 mL to control the indiscriminate spreading of the virus. Plates were incubated for 72 h at 37°C in a 5% carbon dioxide atmosphere. Vero cells were fixed with 10% formalin or 10% formaldehyde in phosphate-buffered saline followed by staining with 1% crystal violet in 50% ethanol or 0.1% crystal violet in PBS. The 50% neutralization concentration (NC_50_) of SARS-CoV-2-specific IgY was determined via the Reed–Muench method [25]. The log IgY concentration was plotted against the percentage of inhibition of each concentration and the NC_50_ was calculated following a nonlinear variable slope equation according to the equation: Y = 100/(1+10ˆ((LogNC50-X)xHillSlope).

### SARS-CoV-2 infection of Ad5-hACE2 mouse model and anti-RBD IgY treatment

Thirty-four 8–10 week old female C57BL/6 mice were anesthetized with ketamine and transduced intranasally with 2.5×10^8^ PFU of Ad5-ACE2 as described elsewhere [26]. Five days post transduction, mice were intranasally administered (using a micropipettor) either 0.25mg of anti-RBD IgY Ab (n = 7) or 0.25mg of IgY antibodies (n = 6) from adjuvant-immunized chickens (non-specific antibodies). Extracted IgY antibodies were diluted in PBS and 50 μL were administered per mouse without further treatment. Four control mice were given an equal volume of normal saline. After two hours, all mice were infected intranasally with SARS-CoV-2 (1 × 10^5^ PFU) in a total volume of 50 μL DMEM. Mice were monitored daily for morbidity (weight loss) and mortality. All work with SARS-CoV-2 was conducted in the University of Iowa Biosafety Level 3 (BSL-3) Laboratory. To obtain virus titers, lungs were harvested from subgroups of 2–4 animals at the indicated time points (2 and 6 days post infection; total of 17 mice) and homogenized into 1 mL of phosphate buffered saline (PBS), using a manual homogenizer. Lung homogenates were aliquoted into micro tubes and kept at -80°C.

### Infection of BALB/c mice with SARS2-N501Y_MA30_ and anti-RBD IgY treatment

Forty-eight male and fifty-four 8–10 week old female BALB/c mice were used in this study. Mice were anesthetized with ketamine-xylazine and infected intranasally with 5,000 PFU of SARS2-N501Y_MA30_ [27] in a total volume of 50 μl of DMEM. Mice were intranasally administered either 0.25mg of anti-RBD IgY Ab, 0.25mg of IgY antibodies from adjuvant-immunized chickens (non-specific antibodies) or mock treated with PBS at either 24-, 12- or 0 hours prior to infection. Antibodies were diluted in PBS up to a 50 μL volume per mouse.

At five days post infection (dpi), 3–5 mice from each group (total of 37 mice) were sacrificed and lungs were harvested to determine viral titers and for histopathological examination. The remaining animals were monitored for 14 days for survival and changes in body weight.

### SARS-CoV-2 plaque assay

Lung homogenate supernatants were serially diluted in DMEM. Vero E6 cells in 12 well plates were inoculated at 37°C in 5% CO_2_ for 1 h with gentle rocking every 15 min. After removing the inoculum, plates were overlaid with 1.2% agarose containing 4% FBS. After further incubation for 2 days, overlays were removed, and plaques were visualized by staining with 0.1% crystal violet. Viral titers were calculated as PFU per milliliter.

### Histopathology

Lungs were perfused with 5–10 mL of PBS; lungs were then removed and fixed with zinc formalin. Fixed tissues were embedded in paraffin, sectioned, and subjected to hematoxylin and eosin staining and examined by a boarded pathologist. In experiments in which mice were infected with mouse-adapted SARS2-N501Y_MA30_, lung tissues were examined in a post-examination method of masking to group assignment [28]. Lung edema scores evaluated based on extent of distribution in lung as previously performed [29].

### Statistical analysis

Data are expressed as means with standard errors. Statistical analyses were performed using Graph Pad Prism 9 software (GraphPad Software Inc., La Jolla, CA, USA). Intergroup comparisons (virus titers in the lungs and body weight curves) were performed using two-way analyses of variance, followed by Bonferroni’s multiple comparisons test. A p-value of <0.05 was considered statistically significant.

## Results

### Isolation and purification of IgY

**Fig 1:**
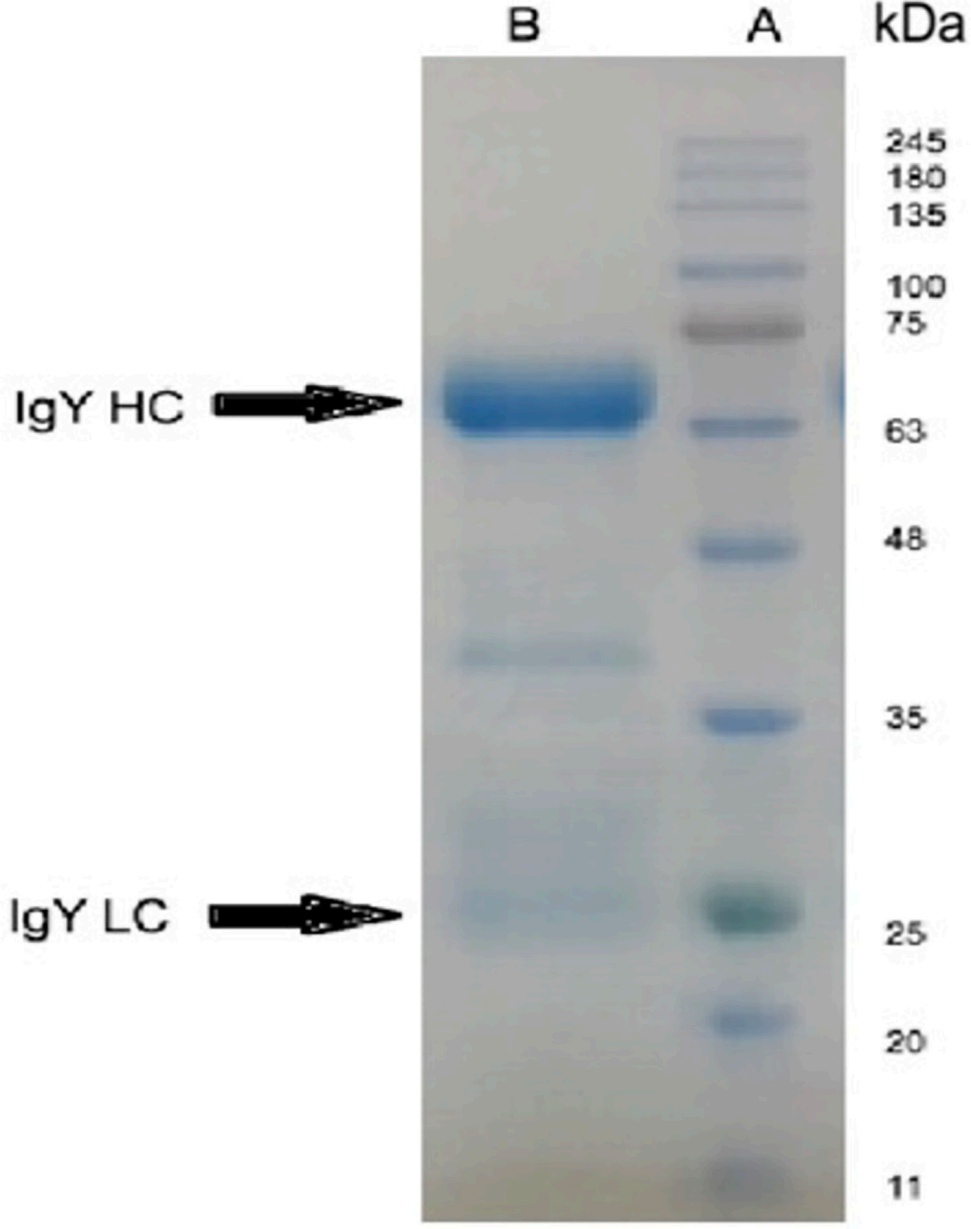
SDS-PAGE profile of the purified IgY antibodies. Lane A: standard protein ladder to indicate molecular mass in kilodaltons (kDa). Lane B: purified IgY with the heavy chain and light chain indicated as HC and LC, respectively.

Total IgY was isolated from eggs of immunized hens. Representative images of SDS-PAGE demonstrate that the IgY disassociated into two band of proteins, a major band at ~68 kDa (heavy chain) and a minor band at ~25 kDa (light chain). A protein band of ~40 kDa (Fig 1), of unknown significance, was also detected. A similarly sized band in IgY preparations was also reported by Dai et al [30].

The yield of each egg yolk after purification was about 12mg/ml with an average volume of approximately 15 mL per each egg yolk. Thus, the total IgY yield per egg was about 180 mg on average.

### Anti-RBD IgY levels from egg yolk

**Fig 2:**
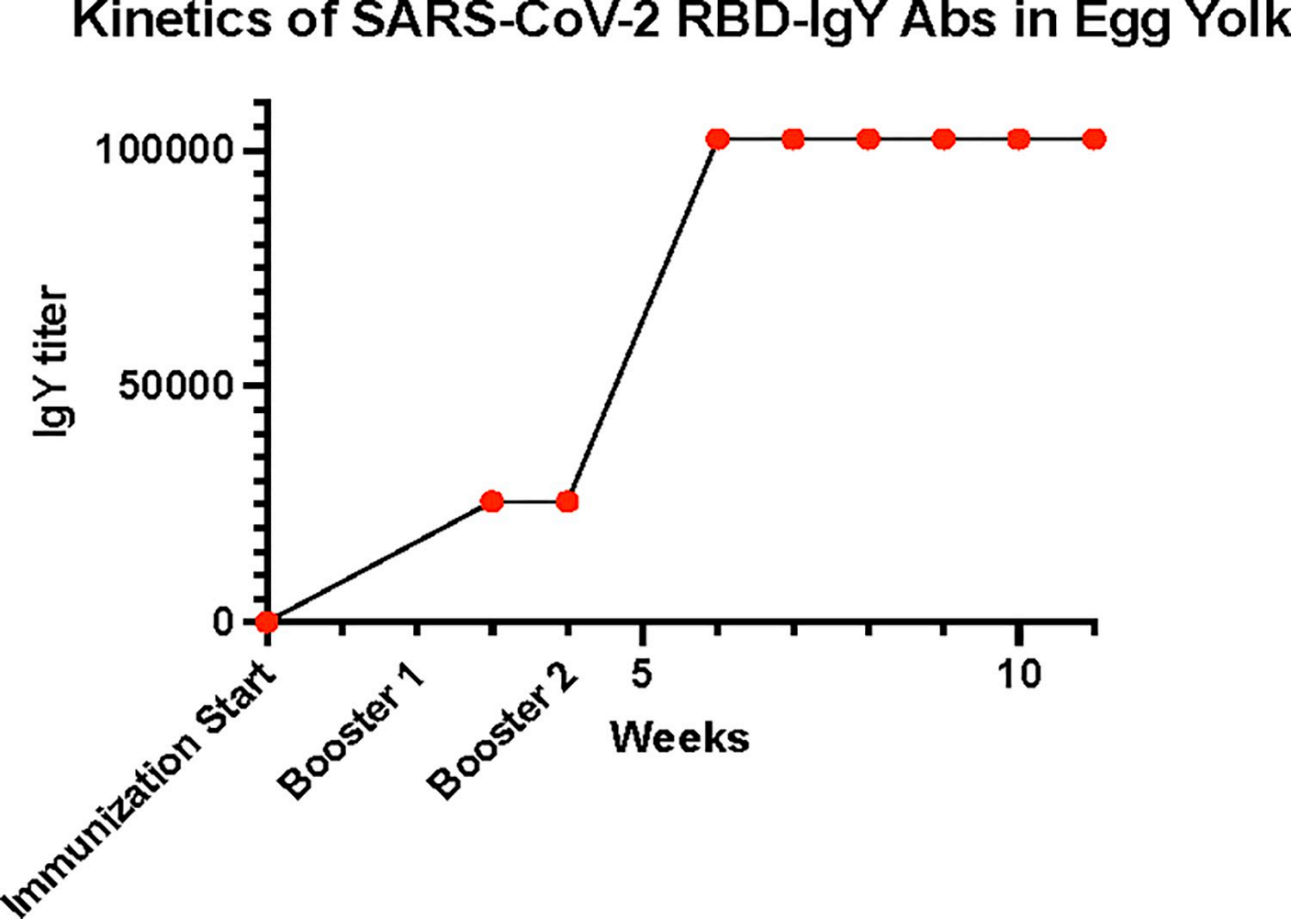
Egg yolk anti-SARS-CoV-2 RBD IgY antibodies were measured after immunization.

Anti-RBD IgY Abs titers were also measured weekly in pooled samples of egg yolks. Low levels of anti-SARS-CoV-2 RBD IgY antibodies were detected in eggs at week 3 after immunization, at a titer of approximately 1x10^4^ binding units, as shown in **Fig 2**. The titers in eggs from immunized hens reached a peak of approximately 1x10^5^ binding units at the 7^th^ week and maintained this level until the 12^th^ week when the experiments were terminated.

### Immunoreactivity of anti-RBD IgY against SARS-CoV-2

**Fig 3:**
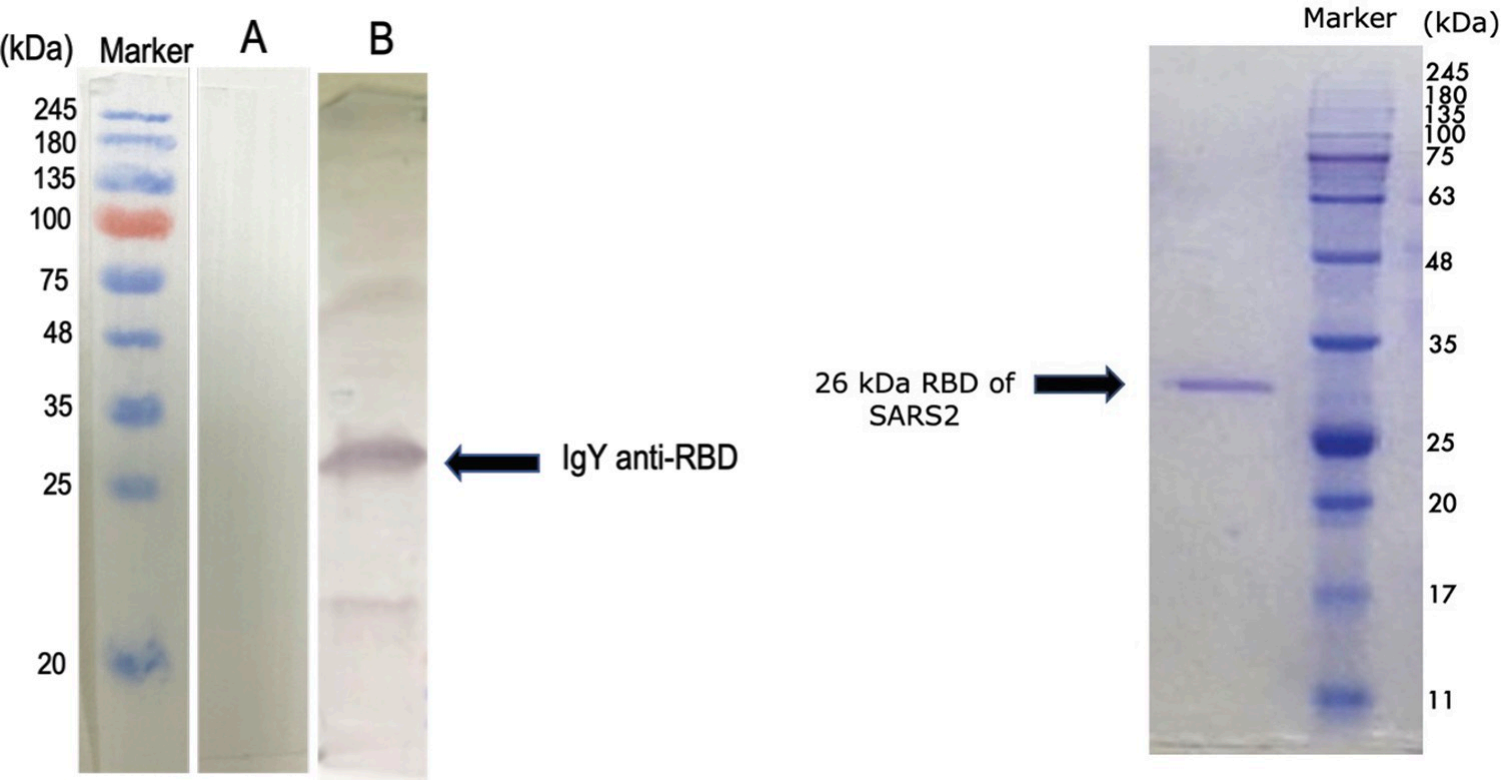
Analyses using western blotting and SDS-PAGE under reducing conditions. Left side is a representative western blot showing IgY from adjuvant-only immunized eggs (Lane A) and from eggs that were immunized with SARS-CoV-2 RBD. The anti-RBD IgY antibody specifically binds to the RBD protein (Lane B). Right side shows the SDS-PAGE of recombinant RBD (Lane C) (molecular weight 26 kDa).

Immunoreactivity of the anti-RBD IgY Abs isolated from egg yolks was assayed using Western Blot analysis. **Fig 3** shows a Western blot with the arrow indicating the 26 kDa RBD protein. The IgY Abs induced by SARS-CoV-2-RBD recognized the 26 kDa RBD recombinant protein when the blot was probed with anti-RBD IgY. These assays show specific anti-RBD IgY antibody binding to the RBD protein.

### IgY Inhibits Virus Replication In Vitro

To further confirm the antiviral activity of RBD-specific IgY, a plaque reduction neutralization assay was performed using SARS-CoV-2 pre-incubated with IgY antibodies before titrating on Vero E6 cells. The IC_50_ of the preparation was 4.44 μg/ml; in contrast, the IgY isolated from adjuvant-injected control chickens demonstrated no neutralization of SARS-CoV-2 infection at concentrations as high as 127.34μg/ml.

### Efficacy of IgY-RBD against Delta and Omicron variants of concern (VOC)

**Fig 4:**
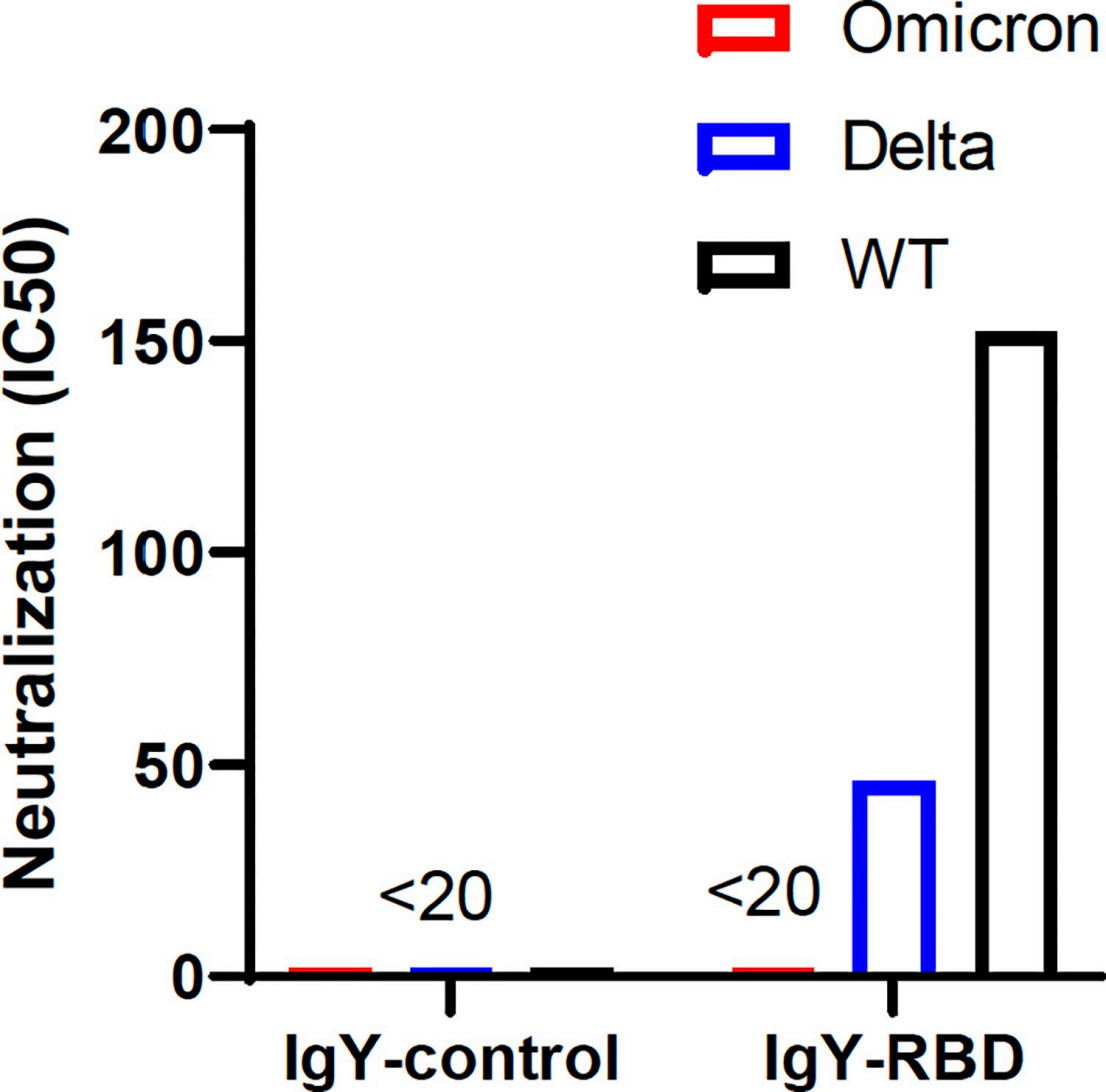
Detection of neutralizing antibody activity against SARS-CoV-2 variants. IgY-control and IgY-RBD antibody titers were measured against 2019-nCoV/USA-WA1/2020 (WT), hCoV-19/USA/MD-HP05647/2021 (Delta) or hCoV19/EHC_C19_2811C (Omicron) in Vero-ACE2/TMPRSS2 cells by plaque reduction neutralization assay. Antibody titers are determined by the highest IgY antibodies dilution that result in a 50% reduction in the number of plaques. LOD: limit of detection, <1:20.

Several SARS-CoV-2 variants have arisen over the past 1–2 years, as the virus continues to evolve, in part in response to widespread vaccination and prior infection. To determine whether IgY-RBD could recognize two of the most recent variants, we infected cells with Delta and Omicron, both of which are VOCs. Consistent with previous reports, effective antibody titers against the Delta variant were decreased 3–4 fold and nearly undetectable when the Omicron variant was assayed (**Fig 4**). The latter result was not surprising since the Omicron RBD is highly mutated compared to ancestral RBDs and Omicron was shown in previous studies to be poorly recognized by antibodies raised against the ancestral strains [31].

### Purified anti-RBD IgY antibodies protect Ad5-ACE2-transduced mice from SARS-CoV-2 infection

**Fig 5:**
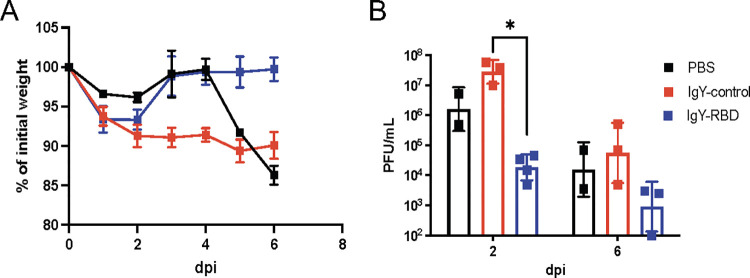
IgY-RBD confers protection in Ad5-hACE2 transduced mice infected with SARS-CoV-2. Mice were transduced with Ad5-hACE2 and treated with IgY-RBD or IgY-control or PBS 2 hours prior to challenge with 2019-nCoV/USA-WA1/2020 SARS-CoV-2. (A) Weights were measured daily. IgY-control (red squares; n = 6), IgY-RBD (blue squares; n = 7) antibodies or PBS (black squares; n = 4) (B) Virus titers in the lungs were measured at the indicated times post infection. Each symbol represents data obtained from one individual mouse. Data in (A) are mean ± s.e.m. Data in (B) are geometric mean ± geometric SD. *P* values determined by two-tailed Student’s t test. Data are from one experiment.

**Fig 6:**
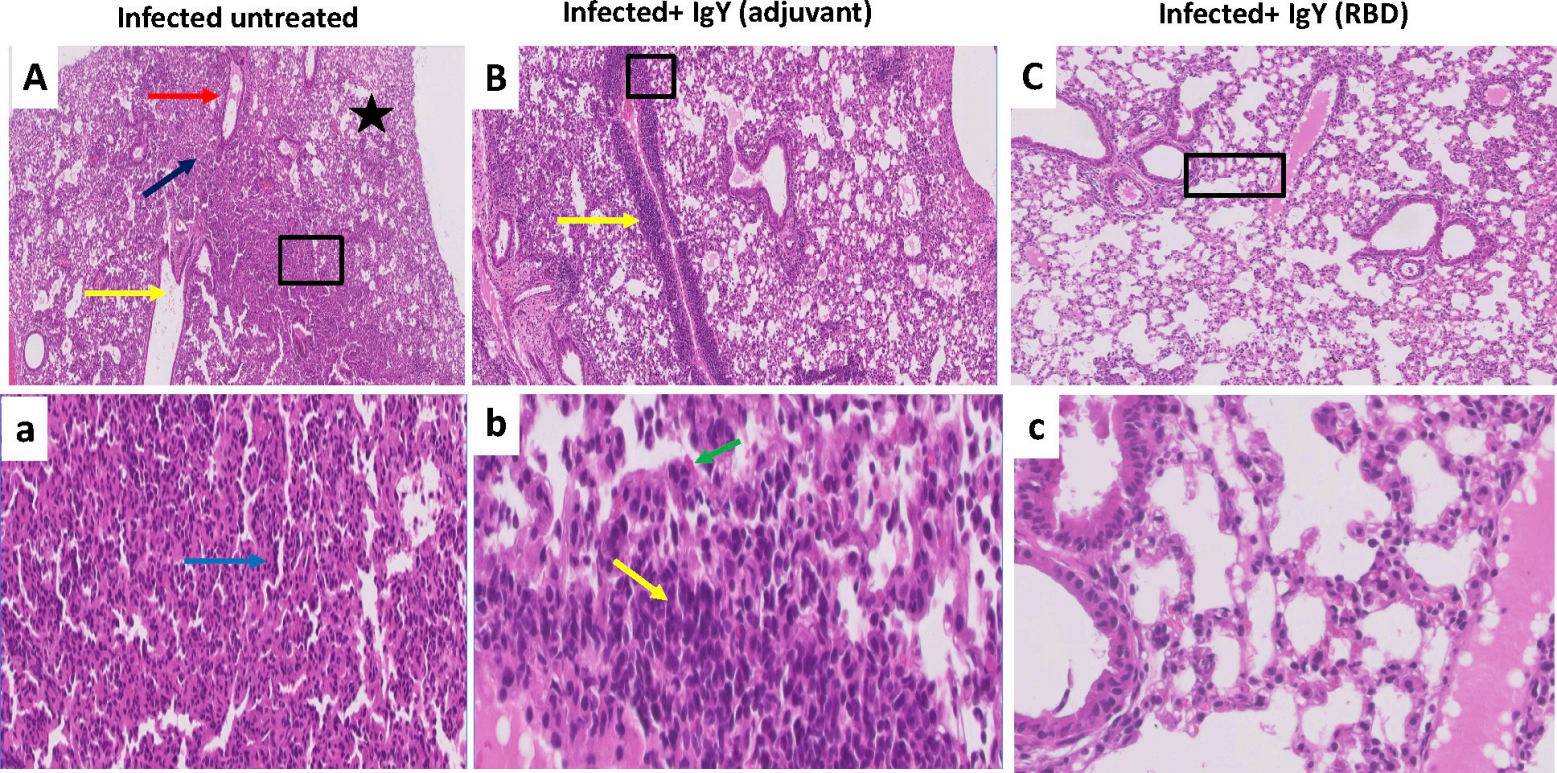
(A and a) Histopathology of the lungs of Ad5-hACE2-transduced mice on day 6 after infection with SARS-CoV-2, showing characteristic bronchioalveolar interstitial pneumonitis with marked alveolar changes represented by alveolar wall thickening due to mononuclear cells infiltration, alveolar pneumocytes type 2 hypertrophic changes and occasionally associated alveolar wall hyalinization (blue arrow). Large number of the alveoli was involved with complete or partial obstruction and presence of some emphysematous alveoli (black star). Some bronchioles appear dilated with presence of exudative fluid in their lumina (yellow arrow). Some blood vessels show partial destruction of their walls with fluid and erythrocytic agglutination (red arrow). (B and b) a perivascular delayed hypersensitive lympho-plasmacytic aggregates with partial vascular wall destruction (yellow arrow) together with moderate alveolitis and alveolar wall thickening and or hyalinization due to pneumocytes hypertrophy and activated alveolar macrophages (green arrow). Some alveoli showed intraluminal aggregates of nonnuclear cells. (C and c), showing apparently normal lung tissue with preserved healthy bronchial tree, alveolar ducts, alveoli besides vascular and stromal structures. Scale bars: 200μm (upper row) and 50μm (lower row).

To examine the efficacy of SARS-CoV-2 IgY antibody *in vivo*, we used two approaches. Mice are naturally resistant to infection with an ancestral strain of SARS-CoV-2, although they can be infected with some of the more recently identified variants of concern (VOC) [32]. For this purpose, we transduced mice with an adenovirus vector expressing human ACE2 (Ad5-hACE2) as described in the Methods. Mice received intranasal administration of 0.25 mg of either anti-RBD IgY antibodies (IgY-RBD group), non-specific IgY (IgY-control group) or PBS, followed by SARS-CoV-2 infection two hours later. Mice in all groups started losing weight from day 1 until day 3 post infection (dpi). Mice receiving the anti-RBD IgY prophylactic treatment recovered quickly and regained their weight as illustrated in **Fig 5B**. The anti-RBD IgY prophylactic treatment group, at both day 2 and day 6 post infection showed reduced viral replication compared with control groups (**Fig 5B**). Histopathological investigation revealed that Ad5-hACE2-transduced mice infected with SARS-CoV-2 showed, bronchioalveolar interstitial pneumonitis with marked alveolar changes represented by alveolar wall thickening due to mononuclear cells infiltration, alveolar pneumocytes type 2 hypertrophic changes and occasionally, associated alveolar wall hyalinization (**Fig 6**). Large numbers of the alveoli were involved, with complete or partial obstruction and presence of emphysematous alveoli. Some bronchioles appeared dilated with presence of exudative fluid in their lumina. Some blood vessels showed partial destruction of their walls with fluid and erythrocytic agglutination. No histopathological improvement in the lungs of non-specific IgY-treated mice was observed compared to the untreated controls, with partial vascular wall destruction together with moderate alveolitis and alveolar wall thickening and or hyalinization due to pneumocyte hypertrophy and activated alveolar macrophages detected. Some alveoli showed intraluminal aggregates of nonnuclear cells. On other hand, normal appearing pulmonary tissue with marked decreased amounts of perivascular and peribranchial inflammatory cell infiltration, hemorrhage, and edema were seen in the group administered anti-RBD IgY antibodies.

### Purified anti-RBD IgY antibodies protect mice from lethal SARS-CoV-2 infection

**Fig 7:**
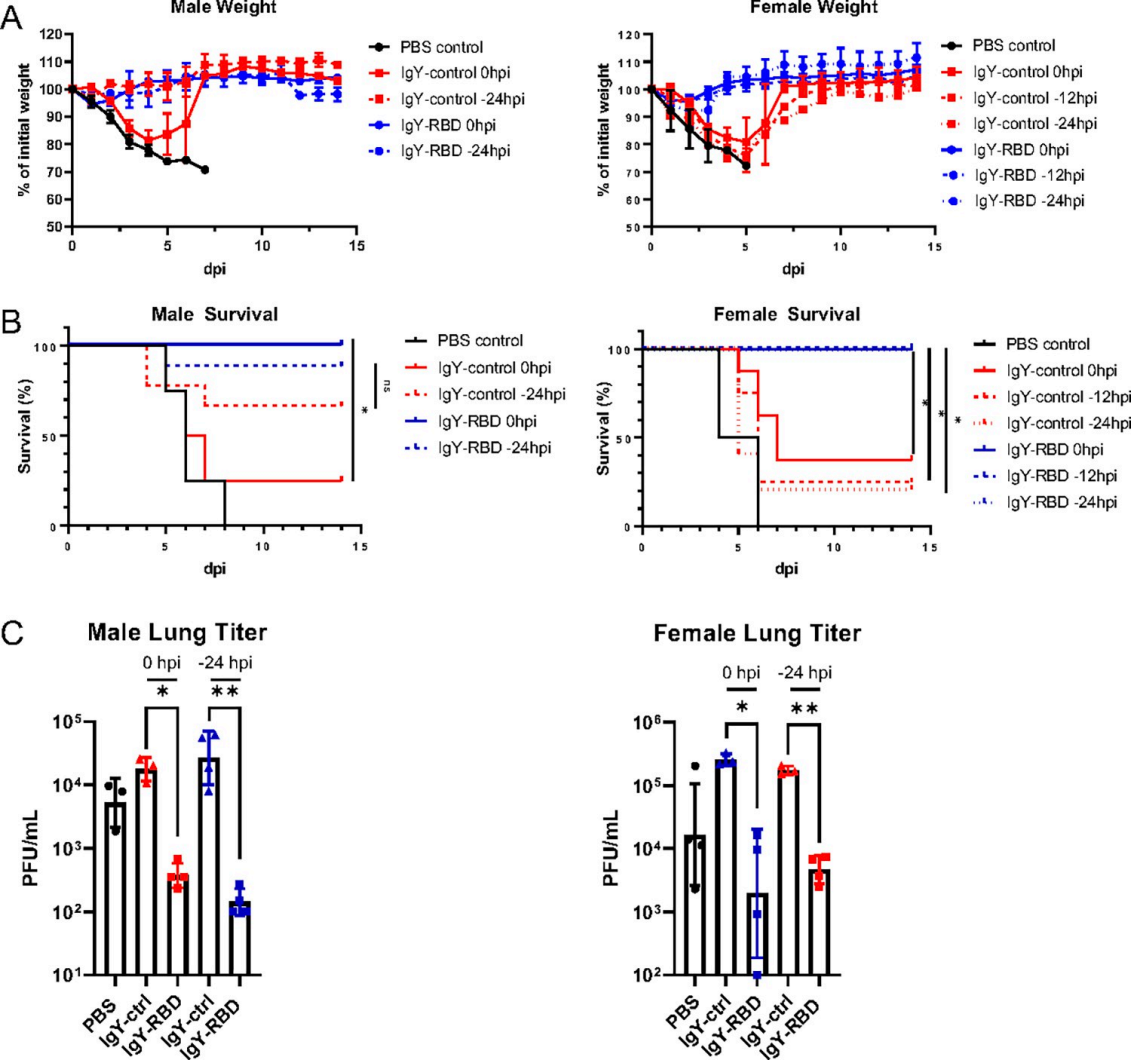
IgY-RBD confers *in vivo* protection in SARS2-N501Y_MA30_-infected BALB/c mice. Male and female mice were treated with IgY-RBD or IgY-control or PBS at the indicated times prior to intranasal challenge with SARS2-N501Y_MA30_. (A, B) Weight (A) and survival (B) were monitored daily. (C) Levels of infectious virus in the lungs were determined at 5 days post infection. (A and B) Male: n = 4 for PBS, IgY-control 0hpi and IgY-RBD 0hpi; n = 9 for IgY-control -24hpi and IgY-RBD -24hpi. Female: n = 4 PBS, IgY-control -12hpi; n = 5 IgY-control -24hpi, IgY-RBD (0hpi, -12hpi, -24hpi); n = 8 IgY-control 0hpi. (C) each symbol represents data obtained from one individual mouse. Data in (A) are mean ± s.e.m. Data in (C) are geometric mean ± geometric SD. *P* values determined by two-tailed Student’s t test. Data are pooled from three independent experiments.

To evaluate the clinical and antiviral activity of anti-RBD IgY antibodies in mice that developed severe clinical disease, we infected mice with mouse-adapted SARS-CoV-2 (SARS2-N501Y_MA30_) as recently described [27]. BALB/c mice infected with 5000 PFU SARS2-N501Y_MA30_ uniformly succumb to the infection. BALB/c mice were intranasally inoculated with either isotype IgY or anti-RBD IgY prior to, or simultaneous with SARS2-N501Y_MA30_ lethal challenge. In initial experiments, we found that female mice treated 12 or 24 hours before infection were similarly protected by IgY-RBD antibodies. Consequently, male mice were only studied after delivery of IgY antibodies at 24 hours prior to SARS-CoV-2 challenge (**Fig 7A and 7B**). Male and female mice treated with anti-RBD IgY at 24, 12 or 0 hours prior to SARS2-N501Y_MA30_ showed reduced weight loss and nearly complete survival. Further examination revealed significantly lower levels of virus in the lungs of anti-RBD-treated mice at 5 dpi (**Fig 7C**).

**Fig 8:**
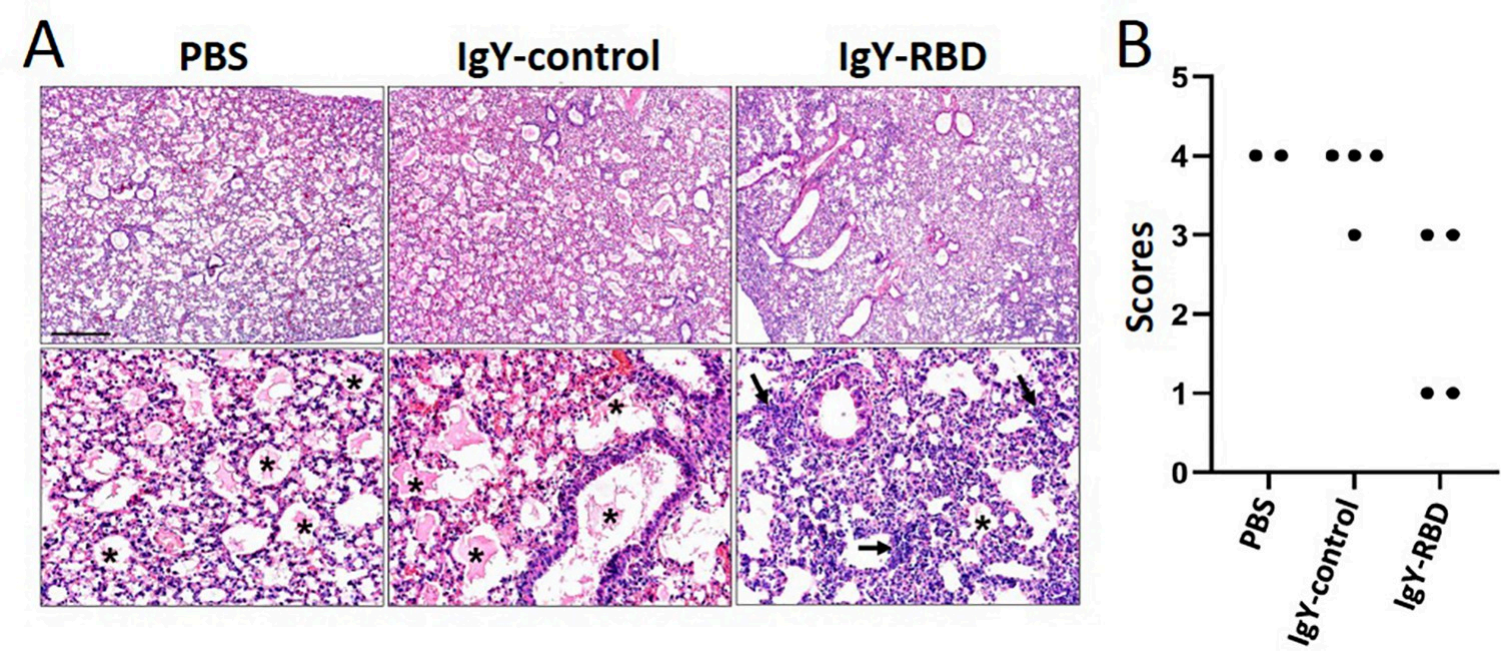
IgY-RBD diminishes pathological changes in mice infected with SARS2-N501Y_MA30_. Lungs were harvested from mice treated with PBS, IgY-control or IgY-RBD at 5dpi. (A) Pulmonary edema (*) was widespread in PBS and IgY-control groups but was detected less in IgY-RBD-treated mice. Increased cellular infiltrates (arrows) in interstitial and perivascular regions were seen in the IgY-RBD group. HE stain, Bar = 480 and 95mm, top and bottom panels, respectively. (B) Ordinal scoring of lung edema. The median scores of the three groups varied significantly (P = 0.0317, Kruskal-Wallis test) and IgY-RBD trended to have reduced edema compared to PBS and IgY-control groups (P = 0.0892 and P = 0.0935, respectively, Dunn’s Posttest).

Lungs were harvested at 5 dpi and analyzed for pathological changes. The IgY-control and PBS groups had widespread edema whereas analysis of the IgY-RBD group revealed scattered localized edema with noted increased detection of cellular infiltration (**Fig 8A**). We scored the lungs for pulmonary edema and tested the null hypothesis that the median scores for the three groups were the same, but we found that the group medians varied significantly (P = 0.0317, Kruskal-Wallis test; **Fig 8B**). Next, we evaluated for group-specific comparisons and IgY-RBD trended to have reduced edema compared to PBS and IgY-control groups (P = 0.0892 and P = 0.0935, respectively, Dunn’s Posttest).

## Discussion

Besides regulatory authorizations or approvals of COVID-19 vaccines, antivirals, and neutralizing monoclonal antibodies in various countries, there remains a global need to develop additional safe, effective, easy-to-produce, and inexpensive treatments to prevent or reduce the risk of acquiring SARS-CoV-2 infection [33,34]. IgY may provide a low cost, safe, and fast approach (produced in 5–8 weeks, including the time required for immunogen preparation) for rapid development of prophylaxis and therapeutic agents against a variety of pathogens [5,8,35]. The use of chickens in the production of these antibodies allows industrial scale-up and its use in many countries worldwide where chicken farming is available.

In this study, hens injected with SARS-CoV-2 RBD subunit protein developed high titers of SARS-CoV-2 RBD-specific IgY Abs in their eggs at 3 weeks post injection and these titers remained at this high titer for 12 weeks. Other studies have shown that hens maintain a high antibody titer against a variety of antigens used for immunization for at least 3 to 4 months [4,19,36].

In Western blot assays, anti-RBD SARS-CoV-2 IgY-Abs exhibited immunoactivity to viral RBD recombinant protein. Targeting RBD in this study to generate IgY antibodies was selected on the basis of reports that showed RBD binding to hACE2 is required for SARS-CoV-2 entry into cells [37]. Moreover, chicken IgY Abs reportedly exhibit higher avidity (10^9^ L/mol) after the first immunization than sheep or other mammals, which must receive four boosters to reach similar avidity values [38]. Plaque reduction neutralization assay data showed that anti-RBD IgY inhibited virus replication in Vero E6 cells with an NC_50_ of 4.44 μg/ml when it was pre-incubated with live SARS-CoV-2.

The potential use of IgY antibodies in MERS-CoV was reported in our previous publications, where we reported the inhibitory effect of IgY antibodies targeting the S1 [4] and the full spike protein [19] of MERS-CoV. IgY antibodies targeting the S1 protein neutralized MERS-CoV in Vero cells and significantly reduced viral antigen expression (p = 0.0196) and markedly reduced inflammation in lung tissue. The second study showed *in vitro* neutralization activity of the anti-S IgY antibodies in Vero cells. As in the study using S1 protein-specific IgY, *in vivo* administration of the anti-S IgY antibody revealed inhibition of virus replication and reduced inflammation, compared to controls. Pathogen-specific IgY antibodies were shown to be highly effective in other bacterial and viral infections of the respiratory system, with no reported side effects [19,39].

Mice are resistant to infection with SARS-CoV-2 but can be rendered susceptible if the human ACE2 receptor is inserted exogenously (by transduction) with a replication-deficient adenovirus (Ad5-hACE2) [26] or if virus is adapted to mice [40–44]. Ad5-hACE2 transduced C57BL/6 mice infected with SARS-CoV-2 or BALB/c mice infected with SARS2-N501Y_MA30_ showed signs of pneumonia characterized by weight loss, severe pulmonary pathology including perivascular to interstitial inflammatory cell infiltrates, necrotic cell debris, and alveolar edema with high-titer virus replication in the lungs. In our study, prophylactic intranasal administration of the SARS-CoV-2 anti-RBD IgY-Abs, conferred protection against viral challenge, nearly complete survival with marked reduction in viral load, recovery of body weight and reduction of lung tissue inflammation. Passive immunoprophylaxis by surface treatment with IgY (e.g., via nasal spray, lozenges, etc.) may provide an effective prophylactic approach to neutralizing pathogens at the earliest stages of infection. This approach may be especially attractive in countries with limited- resource where vaccination is unavailable, where new viral variants may evade immunity produced by vaccines or previous infection, or where immediate immunity is required [8]. A recent study showed that nasal spray of IgY antibodies against RBD trimer vaccine candidate of SARS-CoV-2 that covered all mutations in Alpha/Gamma VOCs and partial mutations in Omicron VOC [45]. The generated IgY-Abs showed a broad prophylactic protection in Ad5-hACE2 transduced C57BL/6 mice infected with high doses of SARS-CoV-2.

Prophylaxis to neutralize the virus before it enters the nasopharynx using IgY against SARS-CoV-2 or MERS-CoV could provide a means to curb the transmission and reduce severity of disease. Lee et al [8] suggested two anti-viral mechanisms when IgY antibodies are delivered intranasally: 1) mucosally administered anti-SARS-CoV-2 IgY may bind to the spike protein on the surface of the virus, competing with the binding of the viral spike protein to hACE2 to prevent cell entry and infection, 2) anti-SARS-CoV-2 IgY may agglutinate SARS-CoV-2 on the mucosal surface, thus preventing viral lateral motility and entry across the mucosa.

A recent study [14] showed the *in vivo* antiviral activity of anti-SARS-CoV-2 IgY antibodies administered via oral spray or nasal drip. These antibodies were remained in the upper airways for hours depending on the administration method used. Another study showed that IgY antibodies raised against the spike glycoprotein of SARS-CoV-2 virus successfully inhibited the critical initial adhesion of viral spike glycoproteins to human ACE2 and inhibited viral replication *in vitro* [46].

Several reports have investigated the use of IgY antibodies in a variety of infections [47]. Nasally administered IgY antibodies were found to prevent mortality in influenza A virus-infected mice with significant reduction in lung pathology when delivered up to 6 hours prior to lethal virus exposure [36,48]. Oral prophylaxis of respiratory infection is another option for administering, which is proposed to function by inhibiting virus colonization and replication in the mucous membranes and reducing their adherence ability; thus preventing damage to the mucosal lining [49]. The oral route was utilized in a long term treatment (up to 12 years) of cystic fibrosis patients with specific IgY against *P*. *aeruginosa* to prevent pulmonary infection [50]; with significant reduction in *P*. *aeruginosa* infections compared with cystic fibrosis control patients, with no adverse events [50]. A similar approach was proposed to block the entry of SARS-CoV-2 virus into the mouth, where it actively replicates, and prevent the development of disease or injury to the lungs [51]. This type of oral treatment would also be very useful in immunocompromised individuals, such as the elderly, those infected with HIV, or those with severe co-morbidities, such as cancer [52–54].

The potential of IgY to address a wide range of common and fatal diseases in both farm animals and humans is gaining more interest [7,55–57] and warrants further exploration against infectious agents, including those associated with pandemics. For example, the availability of COVID-19 vaccines with outstanding safety and efficacy profiles remains limited, with an estimated 65% of the world population have received at least one dose of the vaccine as of April 14, 2022 [58]. A key bottleneck in mRNA (COVID-19) vaccine manufacturing is a global shortage of essential components (such as nucleotides, enzymes, and lipids) [59], which has slowed down production of the estimated 11 billion doses needed to fully vaccinate 70% of the world’s population–the figure assumed needed to reach herd immunity. This shortage especially impacts low- and lower-middle-income countries [59]. In contrast, within 6 weeks of identifying an epidemic or pandemic-causing virus, millions of egg-laying hens immunized throughout the world can provide billions of doses of drug substance, as each egg can produce many doses.

## Conclusions

Our data show specific and efficient neutralization effect of anti-RBD IgY antibodies against SARS-CoV-2 using *in vitro* and in mice infected with SARS-CoV-2. This is the first report that shows the potential use of IgY antibodies as a prophylactic vaccine against SARS-CoV-2. Clinical trials are needed to evaluate the efficacy of anti-RBD IgY antibodies for use as prophylactics against SARS-CoV-2 in humans, especially in high-risk populations with weak immunity such as elderly persons and immunocompromised individuals, or with occupational exposure, such as frontline workers, including those in healthcare. Our results provide a proof of concept for the potential formulation of IgY-Abs as nasal aerosol spray to provide rapid protection in individuals at crowded environments (schools, hospitals, airplanes, etc.) as well as the potential of large scale for global production in the time of a pandemic. Furthermore, It is possible to produce the IgY within 6 weeks hens inoculation in case of emergence of entirely new viral strains appear.
